# {μ-6,6′-Dimeth­oxy-2,2-[propane-1,3-diylbis(nitrilo­methanylyl­idene)]diphenolato}trinitratocopper(II)dysprosium(III) methanol monosolvate

**DOI:** 10.1107/S1600536811005253

**Published:** 2011-02-23

**Authors:** Lili Xu, Hong-Feng Li, Peng Chen, Peng-Fei Yan

**Affiliations:** a School of Chemistry and Materials Science, Heilongjiang University, Harbin 150080, People’s Republic of China

## Abstract

In the title heterodinuclear salen-type complex, [CuDy(C_19_H_20_N_2_O_4_)(NO_3_)_3_]·CH_3_OH, the copper(II) ion is tetra­coordinated by two imino N atoms [Cu—N = 1.961 (4) and 1.968 (4) Å] and two phenolate O atoms [Cu—O = 1.931 (3) and 1.938 (3) Å] in a planar geometry. The ten-coordin­ate Dy^III^ ion is ligated by six O atoms of three nitrate groups and four O atoms from the ligand [Dy—O = 2.368 (3)–2.601 (3) Å]. In the crystal, complex mol­ecules and solvent mol­ecules are linked by inter­molecular O—H⋯O hydrogen bonds.

## Related literature

For similar Cu–*Ln* complexes (*Ln* = lanthanide), see: Bao *et al.* (2010 ▶); Elmali & Elerman (2003 ▶, 2004 ▶); Wang *et al.* (2008 ▶); Xing *et al.* (2008 ▶). For bond-valence calculations, see: Pauling (1947 ▶).
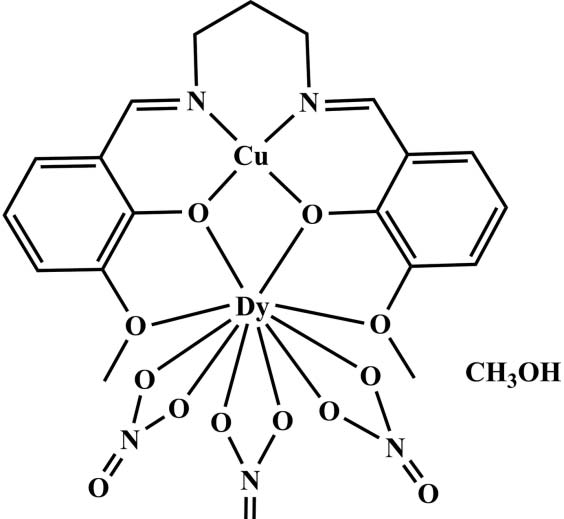


## Experimental

### 

#### Crystal data

[CuDy(C_19_H_20_N_2_O_4_)(NO_3_)_3_]·CH_4_O
*M* *_r_* = 784.49
Triclinic, 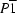

*a* = 8.3572 (17) Å
*b* = 12.130 (2) Å
*c* = 13.891 (3) Å
α = 91.64 (3)°
β = 106.85 (3)°
γ = 99.52 (3)°
*V* = 1324.8 (4) Å^3^
*Z* = 2
Mo *K*α radiation
μ = 3.68 mm^−1^
*T* = 293 K
0.15 × 0.12 × 0.11 mm

#### Data collection

Bruker SMART1000 CCD diffractometer
Absorption correction: multi-scan (*SADABS*; Sheldrick, 2003 ▶) *T*_min_ = 0.595, *T*_max_ = 0.667
13040 measured reflections
6008 independent reflections
5600 reflections with *I* > 2σ(*I*)
*R*_int_ = 0.032

#### Refinement

*R*[*F* ^2^ > 2σ(*F* ^2^)] = 0.039
*wR*(*F* ^2^) = 0.108
*S* = 1.05
6008 reflections
373 parameters
H-atom parameters constrained
Δρ_max_ = 2.37 e Å^−3^
Δρ_min_ = −0.88 e Å^−3^

### 

Data collection: *SMART* (Bruker, 2001 ▶); cell refinement: *SAINT-Plus* (Bruker, 2003 ▶); data reduction: *SAINT-Plus*; program(s) used to solve structure: *SHELXS97* (Sheldrick, 2008 ▶); program(s) used to refine structure: *SHELXL97* (Sheldrick, 2008 ▶); molecular graphics: *SHELXTL* (Sheldrick, 2008 ▶); software used to prepare material for publication: *SHELXL97*.

## Supplementary Material

Crystal structure: contains datablocks global, I. DOI: 10.1107/S1600536811005253/vm2074sup1.cif

Structure factors: contains datablocks I. DOI: 10.1107/S1600536811005253/vm2074Isup2.hkl

Additional supplementary materials:  crystallographic information; 3D view; checkCIF report

## Figures and Tables

**Table 1:** Hydrogen-bond geometry (Å, °)

*D*—H⋯*A*	*D*—H	H⋯*A*	*D*⋯*A*	*D*—H⋯*A*
O1*M*—H4*M*⋯O2^i^	0.89	2.03	2.852 (8)	152

Symmetry code: (i) 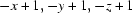.

**Fig. 1.**
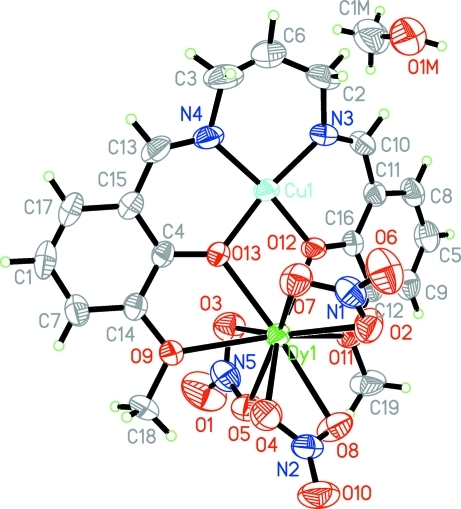
The molecular structure of the title compound, showing 50% probability displacement ellipsoids.

**Fig. 2.**
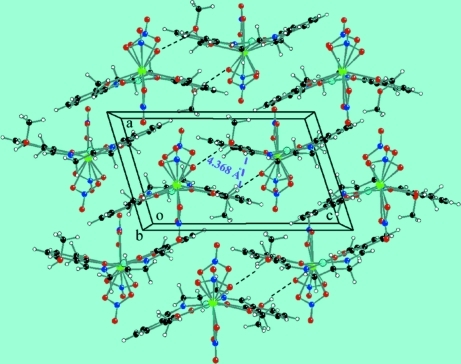
The packing diagram of the title compound along the b direction. The black dotted line shows the H-bonding between the methanol molecules and the complexes. The purple dotted line shows the π-π interaction of the adjacent aromatic rings of the 2-oxy-3-methoxybenzylidene groups [Symmetry code: (i) 1 - x, 1 - y, 1 - z].

## supplementary crystallographic information

### Comment

In continuation of the studies of salen-type lanthanide complexes (Elmali *et al.*, 2003, Elmali *et al.*, 2004), we present here the synthesis and the crystal structure of the title compound (Fig. 1). The Dy^III^ ion is ligated to six oxygen atoms from three bidentate nitrate groups and four oxygen atoms from the ligand, similar to what is found in previously published structures (Wang *et al.*, 2008, Xing *et al.*, 2008, Bao *et al.*, 2010). The Dy—O bond distances are in the range of 2.368 (3) to 2.601 (3) Å, in accordance with the reported values. The planar coordinated Cu(II) ion is tetra-coordinated by two imino nitrogen atoms (Cu—N bond distance range, 1.961 (4)–1.968 (4) Å) and two phenolate oxygen atoms from the ligand (Cu—O bond distance range, 1.931 (3)–1.938 (3) Å). The positive charge of the Dy^III^ and Cu(II) ions are balanced by the ligand *L*^2-^ and three nitrate groups (*L* = *N*,*N*'- bis(2-oxy-3-methoxybenzylidene)-1,3-diaminopropane). However, bond valence calculations (Pauling, 1947) indicate a bond valency of +2 for the Dy ion. This difference is attributed to the longer bond distances of Dy—O. One MeOH molecule is dissociative in the complex, forming H-bonding with the adjacent nitrate group (O···O distance 2.852 (7) Å, Table 1). A methanol molecule is absent in the case of a reported Cu—Eu complex, where a similar coordination environment for Cu(II) and Eu(III) ions could be found (Xing *et al.*, 2008). Furthermore, an acetone molecule is observed instead of the methanol molecule in the case of the reported structures with Sm(III) and Cu(II) ions in a similar coordination environment (Wang *et al.*, 2008). Weak π-π interactions between adjacent aromatic rings of the 2-oxy-3-methoxybenzylidene groups could also be observed (Fig. 2, Cg(5)···Cg(5)^i ^distance = 4.368 (3) Å, Cg(5) is the centroid of ring C5-C16, symmetry code (i): 1 - *x*, 1 - *y*, 1 - *z*).

### Experimental

To a 1:1 MeOH/CH_2_Cl_2_ solution (20 ml) of H_2_L (0.0684 g) and Cu(OAc)_2_.2H_2_O (0.0440 g) was added a MeOH solution (10 ml) of Dy(NO_3_)_3_].6H_2_O (0.0753 g) at the ambient temperature. The color of the solution immediately changed to green. After stirring for 5 hrs, the solution was filtered to remove the suspended particles. Green single crystals suitable for X-ray determination were obtained by slow diffusion of diethylether into the filtrate in one week. [CuDy(C_19_H_20_N_2_O_4_)(NO_3_)_3_].CH_3_OH Elemental Anal. Calc. for C_20_H_24_N_5_O_14_CuDy: C, 30.62; H, 3.08; N, 8.93 wt%, Found: C, 30.61; H, 3.10; N, 8.93 wt%.

### Refinement

H atoms bound to C atoms were placed in calculated positions and treated as riding on their parent atoms, with C—H = 0.93 Å (aromatic C), C—H = 0.97 Å (methylene C), and with *U*_iso_(H) = 1.2Ueq(C) or C—H = 0.96 Å (methyl C) with *U*_iso_(H) = 1.5Ueq(C). The H atom bound to the O atom is found from the Fourier difference map, and refined with *U*_iso_(H) = 1.5Ueq(O).

### Crystal data

**Table:**

[CuDy(C_19_H_20_N_2_O_4_)(NO_3_)_3_]·CH_4_O	*Z* = 2
*M**_r_* = 784.49	*F*(000) = 772
Triclinic, *P*1	*D*_x_ = 1.967 Mg m^−^^3^
Hall symbol: -P 1	Mo *K*α radiation, λ = 0.71073 Å
*a* = 8.3572 (17) Å	Cell parameters from 11901 reflections
*b* = 12.130 (2) Å	θ = 6.2–54.9°
*c* = 13.891 (3) Å	µ = 3.68 mm^−^^1^
α = 91.64 (3)°	*T* = 293 K
β = 106.85 (3)°	Block, green
γ = 99.52 (3)°	0.15 × 0.12 × 0.11 mm
*V* = 1324.8 (4) Å^3^	

### Data collection

**Table:**

Bruker SMART1000 CCD diffractometer	6008 independent reflections
Radiation source: fine-focus sealed tube	5600 reflections with *I* > 2σ(*I*)
graphite	*R*_int_ = 0.032
Detector resolution: 0 pixels mm^-1^	θ_max_ = 27.5°, θ_min_ = 3.1°
ω scans	*h* = −10→10
Absorption correction: multi-scan (*SADABS*; Sheldrick, 2003)	*k* = −15→15
*T*_min_ = 0.595, *T*_max_ = 0.667	*l* = −18→18
13040 measured reflections	

### Refinement

**Table:**

Refinement on *F*^2^	Primary atom site location: structure-invariant direct methods
Least-squares matrix: full	Secondary atom site location: difference Fourier map
*R*[*F*^2^ > 2σ(*F*^2^)] = 0.039	Hydrogen site location: inferred from neighbouring sites
*wR*(*F*^2^) = 0.108	H-atom parameters constrained
*S* = 1.05	*w* = 1/[σ^2^(*F*_o_^2^) + (0.0635*P*)^2^ + 1.7058*P*] where *P* = (*F*_o_^2^ + 2*F*_c_^2^)/3
6008 reflections	(Δ/σ)_max_ = 0.002
373 parameters	Δρ_max_ = 2.37 e Å^−^^3^
0 restraints	Δρ_min_ = −0.88 e Å^−^^3^

### Special details

**Table:**

Geometry. All e.s.d.'s (except the e.s.d. in the dihedral angle between two l.s. planes) are estimated using the full covariance matrix. The cell e.s.d.'s are taken into account individually in the estimation of e.s.d.'s in distances, angles and torsion angles; correlations between e.s.d.'s in cell parameters are only used when they are defined by crystal symmetry. An approximate (isotropic) treatment of cell e.s.d.'s is used for estimating e.s.d.'s involving l.s. planes.
Refinement. Refinement of *F*^2^ against ALL reflections. The weighted *R*-factor *wR* and goodness of fit *S* are based on *F*^2^, conventional *R*-factors *R* are based on *F*, with *F* set to zero for negative *F*^2^. The threshold expression of *F*^2^ > σ(*F*^2^) is used only for calculating *R*-factors(gt) *etc*. and is not relevant to the choice of reflections for refinement. *R*-factors based on *F*^2^ are statistically about twice as large as those based on *F*, and *R*- factors based on ALL data will be even larger.

### Fractional atomic coordinates and isotropic or equivalent isotropic displacement parameters (Å^2^)

**Table:**

	*x*	*y*	*z*	*U*_iso_*/*U*_eq_	
Dy1	0.37849 (2)	0.787569 (14)	0.221621 (13)	0.03841 (9)	
Cu1	0.31877 (7)	0.49734 (4)	0.17449 (4)	0.03874 (13)	
O1	−0.0835 (6)	0.8890 (5)	0.1545 (6)	0.110 (2)	
O2	0.6579 (5)	0.7776 (3)	0.3469 (3)	0.0593 (9)	
O3	0.0638 (5)	0.7570 (3)	0.1705 (3)	0.0595 (9)	
O4	0.5635 (5)	0.9497 (3)	0.1845 (3)	0.0552 (8)	
O5	0.1903 (5)	0.9285 (3)	0.2077 (3)	0.0569 (8)	
O6	0.8516 (6)	0.6889 (5)	0.3236 (4)	0.0776 (13)	
O7	0.6210 (5)	0.6967 (3)	0.2017 (3)	0.0556 (8)	
O8	0.5279 (5)	0.9712 (3)	0.3301 (3)	0.0610 (9)	
O9	0.2663 (5)	0.8246 (3)	0.0324 (2)	0.0473 (7)	
O10	0.6882 (6)	1.1015 (3)	0.2812 (4)	0.0751 (12)	
O11	0.3043 (4)	0.7795 (2)	0.3860 (2)	0.0434 (7)	
O12	0.3359 (4)	0.6077 (2)	0.2818 (2)	0.0394 (6)	
O13	0.2861 (4)	0.6277 (2)	0.0987 (2)	0.0436 (7)	
N1	0.7158 (6)	0.7207 (4)	0.2913 (3)	0.0514 (9)	
N2	0.5956 (5)	1.0105 (3)	0.2662 (3)	0.0471 (9)	
N3	0.3609 (5)	0.3818 (3)	0.2707 (3)	0.0459 (8)	
N4	0.2908 (6)	0.4019 (3)	0.0522 (3)	0.0547 (10)	
N5	0.0505 (6)	0.8594 (4)	0.1763 (4)	0.0601 (11)	
C1	0.0878 (7)	0.6441 (5)	−0.2067 (3)	0.0605 (14)	
H1A	0.0384	0.6485	−0.2754	0.073*	
C2	0.4169 (9)	0.2780 (5)	0.2452 (5)	0.0690 (16)	
H2A	0.5396	0.2931	0.2605	0.083*	
H2B	0.3880	0.2210	0.2880	0.083*	
C3	0.3479 (13)	0.2916 (5)	0.0603 (5)	0.094 (3)	
H3A	0.2784	0.2431	0.0013	0.113*	
H3B	0.4638	0.3036	0.0569	0.113*	
C4	0.2237 (5)	0.6294 (3)	−0.0002 (3)	0.0351 (8)	
C5	0.2281 (7)	0.5542 (5)	0.5486 (3)	0.0544 (12)	
H5A	0.1989	0.5424	0.6076	0.065*	
C6	0.3444 (16)	0.2349 (7)	0.1431 (6)	0.125 (4)	
H6A	0.3942	0.1694	0.1365	0.150*	
H6B	0.2252	0.2072	0.1351	0.150*	
C7	0.1365 (6)	0.7421 (4)	−0.1414 (3)	0.0492 (10)	
H7A	0.1231	0.8114	−0.1664	0.059*	
C8	0.2574 (6)	0.4665 (4)	0.4969 (3)	0.0498 (11)	
H8A	0.2518	0.3962	0.5220	0.060*	
C9	0.2411 (6)	0.6609 (4)	0.5148 (3)	0.0491 (10)	
H9A	0.2222	0.7204	0.5512	0.059*	
C10	0.3379 (6)	0.3874 (4)	0.3574 (4)	0.0466 (10)	
H10A	0.3491	0.3243	0.3936	0.056*	
C11	0.2961 (5)	0.4819 (4)	0.4057 (3)	0.0395 (8)	
C12	0.2829 (5)	0.6775 (4)	0.4259 (3)	0.0380 (8)	
C13	0.2253 (7)	0.4274 (4)	−0.0364 (4)	0.0516 (11)	
H13A	0.2029	0.3709	−0.0877	0.062*	
C14	0.2046 (5)	0.7339 (4)	−0.0394 (3)	0.0389 (8)	
C15	0.1806 (5)	0.5350 (4)	−0.0678 (3)	0.0415 (9)	
C16	0.3044 (5)	0.5882 (3)	0.3686 (3)	0.0356 (8)	
C17	0.1112 (7)	0.5446 (5)	−0.1721 (3)	0.0543 (12)	
H17A	0.0815	0.4811	−0.2171	0.065*	
C18	0.2464 (9)	0.9328 (4)	−0.0057 (4)	0.0643 (15)	
H18A	0.1317	0.9291	−0.0477	0.097*	
H18B	0.3234	0.9528	−0.0446	0.097*	
H18C	0.2709	0.9882	0.0498	0.097*	
C19	0.2824 (9)	0.8746 (4)	0.4419 (5)	0.0668 (16)	
H19A	0.1704	0.8615	0.4495	0.100*	
H19B	0.2966	0.9404	0.4062	0.100*	
H19C	0.3655	0.8854	0.5073	0.100*	
O1M	0.2556 (10)	0.1421 (5)	0.4462 (5)	0.115 (2)	
H4M	0.3167	0.1636	0.5099	0.172*	
C1M	0.0847 (11)	0.1206 (7)	0.4006 (7)	0.105 (3)	
H1M	0.0625	0.1120	0.3287	0.157*	
H2M	0.0358	0.1818	0.4177	0.157*	
H3M	0.0353	0.0528	0.4231	0.157*	

### Atomic displacement parameters (Å^2^)

**Table:**

	*U*^11^	*U*^22^	*U*^33^	*U*^12^	*U*^13^	*U*^23^
Dy1	0.05015 (14)	0.03209 (12)	0.03392 (12)	0.00629 (8)	0.01485 (9)	0.00087 (8)
Cu1	0.0546 (3)	0.0298 (2)	0.0348 (3)	0.0108 (2)	0.0165 (2)	0.00046 (19)
O1	0.061 (3)	0.109 (4)	0.164 (6)	0.040 (3)	0.024 (3)	0.040 (4)
O2	0.061 (2)	0.070 (2)	0.0459 (18)	0.0213 (18)	0.0094 (16)	−0.0059 (17)
O3	0.0519 (19)	0.054 (2)	0.070 (2)	0.0050 (15)	0.0172 (17)	0.0044 (18)
O4	0.063 (2)	0.0533 (19)	0.0505 (19)	0.0018 (16)	0.0226 (16)	0.0083 (16)
O5	0.064 (2)	0.0459 (18)	0.065 (2)	0.0180 (16)	0.0220 (18)	0.0011 (16)
O6	0.059 (2)	0.110 (4)	0.076 (3)	0.038 (2)	0.026 (2)	0.024 (3)
O7	0.062 (2)	0.061 (2)	0.0497 (19)	0.0150 (17)	0.0242 (16)	−0.0034 (16)
O8	0.069 (2)	0.0489 (19)	0.063 (2)	−0.0089 (16)	0.0290 (19)	−0.0124 (17)
O9	0.072 (2)	0.0358 (15)	0.0326 (14)	0.0105 (14)	0.0122 (14)	0.0045 (12)
O10	0.078 (3)	0.0404 (19)	0.099 (3)	−0.0094 (17)	0.025 (2)	0.005 (2)
O11	0.0644 (19)	0.0357 (14)	0.0360 (14)	0.0111 (13)	0.0237 (14)	−0.0009 (12)
O12	0.0618 (18)	0.0290 (13)	0.0321 (13)	0.0101 (12)	0.0203 (13)	0.0036 (11)
O13	0.0654 (19)	0.0356 (14)	0.0286 (13)	0.0105 (13)	0.0114 (13)	0.0007 (11)
N1	0.055 (2)	0.053 (2)	0.052 (2)	0.0130 (18)	0.0211 (19)	0.0150 (19)
N2	0.048 (2)	0.0328 (17)	0.060 (2)	0.0078 (15)	0.0148 (18)	0.0045 (17)
N3	0.058 (2)	0.0315 (17)	0.049 (2)	0.0156 (15)	0.0133 (17)	0.0027 (15)
N4	0.089 (3)	0.0335 (18)	0.047 (2)	0.0142 (19)	0.029 (2)	−0.0020 (16)
N5	0.058 (3)	0.066 (3)	0.062 (3)	0.022 (2)	0.020 (2)	0.016 (2)
C1	0.059 (3)	0.087 (4)	0.028 (2)	0.015 (3)	0.0008 (19)	−0.002 (2)
C2	0.098 (4)	0.048 (3)	0.071 (3)	0.041 (3)	0.024 (3)	0.010 (3)
C3	0.181 (9)	0.050 (3)	0.073 (4)	0.048 (4)	0.057 (5)	−0.001 (3)
C4	0.0370 (18)	0.040 (2)	0.0286 (17)	0.0037 (15)	0.0121 (14)	−0.0011 (15)
C5	0.064 (3)	0.068 (3)	0.037 (2)	0.012 (2)	0.024 (2)	0.015 (2)
C6	0.211 (11)	0.080 (5)	0.075 (5)	0.091 (6)	−0.004 (6)	−0.018 (4)
C7	0.051 (2)	0.059 (3)	0.037 (2)	0.017 (2)	0.0081 (18)	0.010 (2)
C8	0.056 (3)	0.055 (3)	0.040 (2)	0.010 (2)	0.015 (2)	0.017 (2)
C9	0.054 (3)	0.065 (3)	0.034 (2)	0.017 (2)	0.0191 (19)	0.004 (2)
C10	0.053 (2)	0.037 (2)	0.048 (2)	0.0119 (18)	0.010 (2)	0.0129 (19)
C11	0.042 (2)	0.039 (2)	0.0346 (19)	0.0056 (16)	0.0091 (16)	0.0069 (16)
C12	0.042 (2)	0.044 (2)	0.0307 (18)	0.0092 (16)	0.0134 (16)	0.0054 (16)
C13	0.069 (3)	0.043 (2)	0.044 (2)	0.001 (2)	0.025 (2)	−0.012 (2)
C14	0.040 (2)	0.044 (2)	0.0331 (19)	0.0070 (16)	0.0121 (16)	0.0012 (16)
C15	0.042 (2)	0.044 (2)	0.036 (2)	0.0021 (17)	0.0110 (16)	−0.0042 (17)
C16	0.0358 (18)	0.041 (2)	0.0292 (17)	0.0069 (15)	0.0087 (14)	0.0051 (15)
C17	0.057 (3)	0.066 (3)	0.032 (2)	0.002 (2)	0.0067 (19)	−0.011 (2)
C18	0.101 (4)	0.036 (2)	0.053 (3)	0.015 (2)	0.016 (3)	0.016 (2)
C19	0.110 (5)	0.044 (3)	0.063 (3)	0.017 (3)	0.052 (3)	−0.005 (2)
O1M	0.160 (6)	0.098 (4)	0.085 (4)	0.036 (4)	0.025 (4)	0.015 (3)
C1M	0.084 (5)	0.085 (5)	0.123 (7)	0.021 (4)	−0.007 (5)	0.013 (5)

### Geometric parameters (Å, °)

**Table:**

Dy1—O12	2.368 (3)	C2—C6	1.417 (9)
Dy1—O13	2.414 (3)	C2—H2A	0.9700
Dy1—O4	2.458 (3)	C2—H2B	0.9700
Dy1—O3	2.477 (4)	C3—C6	1.361 (11)
Dy1—O5	2.483 (3)	C3—H3A	0.9700
Dy1—O2	2.499 (4)	C3—H3B	0.9700
Dy1—O11	2.534 (3)	C4—C15	1.389 (6)
Dy1—O7	2.539 (4)	C4—C14	1.410 (6)
Dy1—O8	2.567 (4)	C5—C8	1.360 (7)
Dy1—O9	2.601 (3)	C5—C9	1.387 (7)
Dy1—N5	2.914 (5)	C5—H5A	0.9300
Dy1—Cu1	3.4884 (9)	C6—H6A	0.9700
Cu1—O12	1.931 (3)	C6—H6B	0.9700
Cu1—O13	1.938 (3)	C7—C14	1.378 (6)
Cu1—N4	1.961 (4)	C7—H7A	0.9300
Cu1—N3	1.968 (4)	C8—C11	1.405 (6)
O1—N5	1.190 (7)	C8—H8A	0.9300
O2—N1	1.266 (6)	C9—C12	1.389 (6)
O3—N5	1.268 (6)	C9—H9A	0.9300
O4—N2	1.270 (5)	C10—C11	1.452 (6)
O5—N5	1.272 (6)	C10—H10A	0.9300
O6—N1	1.224 (6)	C11—C16	1.401 (6)
O7—N1	1.261 (6)	C12—C16	1.392 (6)
O8—N2	1.250 (5)	C13—C15	1.460 (7)
O9—C14	1.387 (5)	C13—H13A	0.9300
O9—C18	1.446 (5)	C15—C17	1.413 (6)
O10—N2	1.213 (5)	C17—H17A	0.9300
O11—C12	1.376 (5)	C18—H18A	0.9600
O11—C19	1.438 (5)	C18—H18B	0.9600
O12—C16	1.327 (5)	C18—H18C	0.9600
O13—C4	1.322 (5)	C19—H19A	0.9600
N3—C10	1.275 (6)	C19—H19B	0.9600
N3—C2	1.481 (6)	C19—H19C	0.9600
N4—C13	1.264 (7)	O1M—C1M	1.361 (10)
N4—C3	1.490 (7)	O1M—H4M	0.8898
C1—C17	1.335 (8)	C1M—H1M	0.9600
C1—C7	1.402 (8)	C1M—H2M	0.9600
C1—H1A	0.9300	C1M—H3M	0.9600
			
O12—Dy1—O13	62.51 (10)	O7—N1—O2	115.1 (4)
O12—Dy1—O4	151.34 (13)	O10—N2—O8	122.1 (4)
O13—Dy1—O4	116.69 (12)	O10—N2—O4	121.5 (4)
O12—Dy1—O3	83.06 (12)	O8—N2—O4	116.5 (4)
O13—Dy1—O3	74.94 (13)	C10—N3—C2	114.6 (4)
O4—Dy1—O3	125.32 (13)	C10—N3—Cu1	123.8 (3)
O12—Dy1—O5	126.44 (12)	C2—N3—Cu1	121.6 (3)
O13—Dy1—O5	117.91 (12)	C13—N4—C3	115.8 (4)
O4—Dy1—O5	80.64 (13)	C13—N4—Cu1	124.1 (3)
O3—Dy1—O5	51.28 (13)	C3—N4—Cu1	120.1 (4)
O12—Dy1—O2	75.83 (13)	O1—N5—O3	122.4 (6)
O13—Dy1—O2	111.10 (12)	O1—N5—O5	122.2 (6)
O4—Dy1—O2	78.57 (13)	O3—N5—O5	115.4 (4)
O3—Dy1—O2	151.06 (14)	O1—N5—Dy1	177.9 (5)
O5—Dy1—O2	130.98 (13)	O3—N5—Dy1	57.6 (2)
O12—Dy1—O11	63.88 (10)	O5—N5—Dy1	57.8 (2)
O13—Dy1—O11	120.64 (10)	C17—C1—C7	121.2 (4)
O4—Dy1—O11	122.51 (11)	C17—C1—H1A	119.4
O3—Dy1—O11	75.22 (13)	C7—C1—H1A	119.4
O5—Dy1—O11	77.04 (12)	C6—C2—N3	114.3 (5)
O2—Dy1—O11	77.78 (12)	C6—C2—H2A	108.7
O12—Dy1—O7	74.46 (12)	N3—C2—H2A	108.7
O13—Dy1—O7	66.68 (12)	C6—C2—H2B	108.7
O4—Dy1—O7	79.32 (13)	N3—C2—H2B	108.7
O3—Dy1—O7	141.16 (13)	H2A—C2—H2B	107.6
O5—Dy1—O7	158.96 (13)	C6—C3—N4	118.6 (6)
O2—Dy1—O7	50.09 (12)	C6—C3—H3A	107.7
O11—Dy1—O7	119.63 (11)	N4—C3—H3A	107.7
O12—Dy1—O8	125.97 (12)	C6—C3—H3B	107.7
O13—Dy1—O8	166.55 (12)	N4—C3—H3B	107.7
O4—Dy1—O8	50.43 (12)	H3A—C3—H3B	107.1
O3—Dy1—O8	114.80 (13)	O13—C4—C15	124.0 (4)
O5—Dy1—O8	67.16 (13)	O13—C4—C14	117.9 (4)
O2—Dy1—O8	65.33 (14)	C15—C4—C14	118.1 (4)
O11—Dy1—O8	72.08 (11)	C8—C5—C9	121.2 (4)
O7—Dy1—O8	104.04 (13)	C8—C5—H5A	119.4
O12—Dy1—O9	123.21 (10)	C9—C5—H5A	119.4
O13—Dy1—O9	62.27 (10)	C3—C6—C2	126.5 (9)
O4—Dy1—O9	70.15 (12)	C3—C6—H6A	105.7
O3—Dy1—O9	71.10 (13)	C2—C6—H6A	105.7
O5—Dy1—O9	71.82 (12)	C3—C6—H6B	105.7
O2—Dy1—O9	137.45 (12)	C2—C6—H6B	105.7
O11—Dy1—O9	143.81 (11)	H6A—C6—H6B	106.1
O7—Dy1—O9	95.24 (12)	C14—C7—C1	118.8 (5)
O8—Dy1—O9	110.78 (12)	C14—C7—H7A	120.6
O12—Dy1—N5	105.58 (13)	C1—C7—H7A	120.6
O13—Dy1—N5	96.16 (14)	C5—C8—C11	120.2 (4)
O4—Dy1—N5	102.99 (14)	C5—C8—H8A	119.9
O3—Dy1—N5	25.60 (13)	C11—C8—H8A	119.9
O5—Dy1—N5	25.70 (13)	C5—C9—C12	119.0 (4)
O2—Dy1—N5	148.84 (13)	C5—C9—H9A	120.5
O11—Dy1—N5	75.38 (13)	C12—C9—H9A	120.5
O7—Dy1—N5	161.04 (14)	N3—C10—C11	126.9 (4)
O8—Dy1—N5	91.33 (15)	N3—C10—H10A	116.5
O9—Dy1—N5	68.55 (13)	C11—C10—H10A	116.5
O12—Dy1—Cu1	31.77 (7)	C16—C11—C8	119.4 (4)
O13—Dy1—Cu1	32.27 (7)	C16—C11—C10	122.6 (4)
O4—Dy1—Cu1	135.29 (9)	C8—C11—C10	117.8 (4)
O3—Dy1—Cu1	84.41 (9)	O11—C12—C9	124.6 (4)
O5—Dy1—Cu1	135.66 (9)	O11—C12—C16	114.4 (3)
O2—Dy1—Cu1	87.77 (10)	C9—C12—C16	121.0 (4)
O11—Dy1—Cu1	95.00 (7)	N4—C13—C15	127.7 (4)
O7—Dy1—Cu1	59.94 (9)	N4—C13—H13A	116.2
O8—Dy1—Cu1	151.81 (10)	C15—C13—H13A	116.2
O9—Dy1—Cu1	94.54 (8)	C7—C14—O9	124.6 (4)
N5—Dy1—Cu1	109.96 (11)	C7—C14—C4	121.3 (4)
O12—Cu1—O13	79.77 (12)	O9—C14—C4	114.1 (3)
O12—Cu1—N4	171.66 (15)	C4—C15—C17	119.9 (4)
O13—Cu1—N4	91.99 (15)	C4—C15—C13	121.8 (4)
O12—Cu1—N3	91.17 (14)	C17—C15—C13	117.7 (4)
O13—Cu1—N3	170.84 (14)	O12—C16—C12	118.6 (4)
N4—Cu1—N3	97.10 (17)	O12—C16—C11	122.4 (4)
O12—Cu1—Dy1	40.19 (8)	C12—C16—C11	119.0 (4)
O13—Cu1—Dy1	41.67 (9)	C1—C17—C15	120.6 (5)
N4—Cu1—Dy1	132.12 (12)	C1—C17—H17A	119.7
N3—Cu1—Dy1	129.23 (11)	C15—C17—H17A	119.7
N1—O2—Dy1	98.3 (3)	O9—C18—H18A	109.5
N5—O3—Dy1	96.8 (3)	O9—C18—H18B	109.5
N2—O4—Dy1	98.9 (3)	H18A—C18—H18B	109.5
N5—O5—Dy1	96.5 (3)	O9—C18—H18C	109.5
N1—O7—Dy1	96.5 (3)	H18A—C18—H18C	109.5
N2—O8—Dy1	94.2 (3)	H18B—C18—H18C	109.5
C14—O9—C18	115.2 (4)	O11—C19—H19A	109.5
C14—O9—Dy1	118.9 (2)	O11—C19—H19B	109.5
C18—O9—Dy1	125.6 (3)	H19A—C19—H19B	109.5
C12—O11—C19	116.7 (3)	O11—C19—H19C	109.5
C12—O11—Dy1	118.6 (2)	H19A—C19—H19C	109.5
C19—O11—Dy1	124.8 (3)	H19B—C19—H19C	109.5
C16—O12—Cu1	126.8 (3)	C1M—O1M—H4M	131.5
C16—O12—Dy1	124.4 (2)	O1M—C1M—H1M	109.5
Cu1—O12—Dy1	108.04 (12)	O1M—C1M—H2M	109.5
C4—O13—Cu1	127.3 (3)	H1M—C1M—H2M	109.5
C4—O13—Dy1	126.6 (3)	O1M—C1M—H3M	109.5
Cu1—O13—Dy1	106.06 (12)	H1M—C1M—H3M	109.5
O6—N1—O7	122.9 (5)	H2M—C1M—H3M	109.5
O6—N1—O2	121.9 (5)		

### Hydrogen-bond geometry (Å, °)

**Table:**

*D*—H···*A*	*D*—H	H···*A*	*D*···*A*	*D*—H···*A*
O1M—H4M···O2^i^	0.89	2.03	2.852 (8)	152

Symmetry codes: (i) −*x*+1, −*y*+1, −*z*+1.
